# B7-H3: A Useful Emerging Diagnostic Marker for Colon Cancer

**DOI:** 10.1155/2022/1523338

**Published:** 2022-12-27

**Authors:** Ozgur Bostanci, Pinar Sayin, Remzi Kiziltan, Sermin Algul, Mehmet Akif Aydin, Ozgur Kemik

**Affiliations:** ^1^Department of General Surgery, University of Health Sciences, Seyrantepe Hamidiye Etfal Training and Research Hospital, Istanbul, Turkey; ^2^Department of Anesthesia and Reanimation, University of Health Sciences, Seyrantepe Hamidiye Etfal Training and Research Hospital, Istanbul, Turkey; ^3^Department of General Surgery, Van Yuzuncu Yıl University Faculty of Medicine, Van, Turkey; ^4^Department of Physiology, Van Yuzuncu Yıl University Faculty of Medicine, Van, Turkey; ^5^Department of General Surgery, Altinbas University Bahcelievler Medical Park Hospital, Istanbul, Turkey

## Abstract

**Background:**

Colon cancer is the second most common malignancy causing the majority of cancer-related deaths. B7-H3 concentrations have drawn major interest as possible diagnostic biomarkers of cancer. The aim of this study was to measure the preoperative serum B7-H3 levels and to determine those that are replaced in colon cancer.

**Method:**

We measured preoperative serum B7-H3 concentrations of 90 patients aged 57-69 years diagnosed with colon cancer and 50 age-matched healthy individuals. B7-H3 levels were determined using the sandwich enzyme-linked immunosorbent assay (ELISA). Patients were categorized by stage based on the TNM staging system, and the serum levels of B7-H3 were compared between patients with different TNM stages.

**Result:**

No statistically significant difference was found between the patient and control groups in terms of age and gender. Preoperative serum B7-H3 levels were statistically significantly higher in patients with colon cancer than in the healthy group (*p* < 0.001). Preoperative serum B7-H3 concentration of the stage IV patients was significantly higher than those of the patients with stage I and stage II disease. In addition, higher serum B7-H3 levels were associated with lymph node status and distant metastasis in colon cancer.

**Conclusion:**

We showed that B7-H3 is highly expressed in colon cancer and can be used as a candidate diagnostic biomarker and a potential target in colon cancer in future.

## 1. Introduction

Colon cancer is the second most common major malignancy in women and the third in men, accounting for 10% of all new cancer cases worldwide [1]. Many patients diagnosed with colon cancer are older than 50 years of age, although it can be diagnosed at any age. In several part of world, the incidence of colon cancer has risen in individuals below 50 years of age [2]. The incidence of colon cancer increases by 1.0%-2.4% annually [3]. Colon cancer is a slow evolving disease, which becomes symptomatic when it progresses to advanced stages, and thus, early diagnosis is the most efficient way to prevent colon cancer, which helps specify and remove polyps before turning into cancer [4]. However, there are generally no symptoms in the early stage of the disease, and the rate of patients with early cancer is low [5, 6]. Therefore, exploring highly predictive markers for an early diagnosis of colon cancer has a strong clinical importance for the prevention and treatment of the disease. Survival from colon cancer is significantly influenced by the disease stage at presentation, but the majority of patients present with advanced disease and the survival rate are low in the case of metastasis [7]. Due to the numerous contributing factors in the development of colon cancer, its pathogenesis remains unclear. Thus, the development of novel therapeutic strategies is an important focus in colon cancer investigation. Numerous biomarkers have been suggested for various types of malignancies, including colon cancer. Some of these markers are being used in practice, while others are under development, and the need for novel markers is continuing.

B7-H3 is a member of the B7 superfamily ligands, which are substantial accessorial molecules, and has costimulatory or coinhibitory effects on T cell responses [8–10]. In humans, B7-H3 is located on chromosome 15. Studies have described B7-H3 as a T cell inhibitor that promotes tumor proliferation and aggressiveness [11]. Thus, B7-H3 may be an important target in cancer, and there is a need for studies to further elaborate the role of its expression and regulation in pathogenesis of various cancers.

So far, B7-H3 has been studied in melanoma, prostate cancer, leptomeningeal cancer, neuroblastoma, and sarcoma [12]. However, there is no study in the literature investigating the contribution of B7-H3 levels to the promotion of tumor progression between colon cancers with different locations. Therefore, the objective of this study was to investigate preoperative serum levels of B7-H3 in colon cancer and its correlation with disease progression and circulating tumor cells.

## 2. Materials and Methods

### 2.1. Study Design

This study was designed as a prospective study and conducted in Van Yuzuncu Yil University Hospital.

### 2.2. Patients and Control Group

Ninety patients aged 57-69 years, diagnosed with colon cancer and followed-up in our clinic and age-matched 50 healthy individuals as the control group, were included in this study. All patients with colon cancer were diagnosed by histological examination, and the healthy individuals were enrolled during routine health check-ups served at our hospital. Patients who refused participation in the study were excluded.

Participants' demographic characteristics such as age and gender were recorded. Patients were categorized by stage based on the TNM staging system published by the American Joint Committee on Cancer [13].

### 2.3. B7-H3 Assay

Each blood sample was collected with a vacuum blood collection tube 3 days before surgery. They were immediately centrifuged at 1500 × g for 10 minutes; then, the supernatants were allotted in 1.5 mL tubes (Eppendorf) and stored at -80°C until analysis.

Serum B7-H3 concentration was measured according to the ELISA kit (R&D Systems; Catalog No: DB7H30; solid phase sandwich ELISA; sensitivity: 0.274 ng/mL; assay range: 0.8-50 ng/mL) in line with the instructions of the manufacturer.

### 2.4. Ethics Considerations

Before starting the study, the study protocol was approved by the local ethics committee of Van Yuzuncu Yil University Non-Interventional Clinical Research Ethics Committee (No: 2022/01-07). All participants were informed about the objectives of the study and gave signed written informed consent. The study was conducted in accordance with the Declaration of Helsinki.

### 2.5. Statistical Analysis

Normality of the study variables was checked by performing Shapiro Wilk and single-sample Kolmogorov-Smirnov tests, histograms, Q-Q plots, and box plot graphs. The study variables were reported as mean, standard deviation, minimum, maximum, frequency, and percentage. Variables with two categories were analyzed with the Mann–Whitney *U* test. Variables with 3 or more categories were compared with the Kruskal-Wallis one-way analysis of variance. Multiple comparisons were made with Dunn's test. Nominal variables were evaluated with the Chi-square test with Yates correction. The limit of significance was taken as *p* < 0.05 and two-sided. All analyses were performed using the NCSS 10 (2015. Kaysville, Utah, USA) statistical software.

## 3. Results

The mean age of the patients with colon cancer was 62.54 ± 2.94 years. Among these patients, 52.2% were male (*n* = 47), and 47.8% were female (*n* = 43). The mean age of the control group was 63.54 ± 2.94 years. Among them, 50% were male (*n* = 25), and 50% were female (*n* = 25).  Table 1 shows demographic characteristics of the patient and control groups.

**Figure 1:**
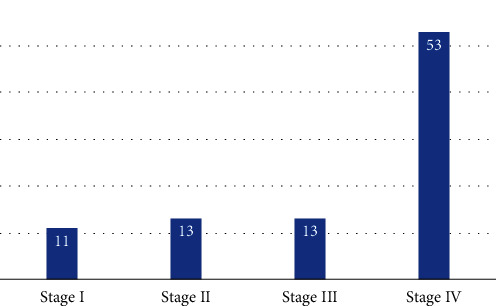
Distribution of the patients according to TNM staging.

According to the TNM staging system, there were 11 patients in stage I, 13 patients in stage II, 13 patients in stage III, and 53 patients in stage IV (Figure 1).

**Figure 2:**
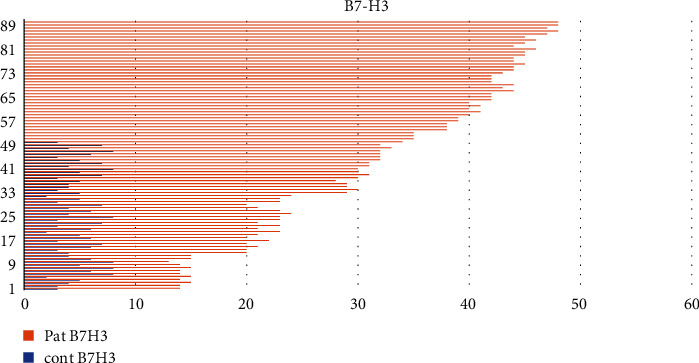
The histogram of the B7-H3 levels of all groups.

The preoperative level of B7-H3 was found as 4.91 ± 1.83 (min–max: 2-8) ng/mL in the control group and 31.81 ± 11.02 (min–max: 13-48) in the patient group.  Figure 2 shows B7-H3 of the patient and control groups.

**Figure 3:**
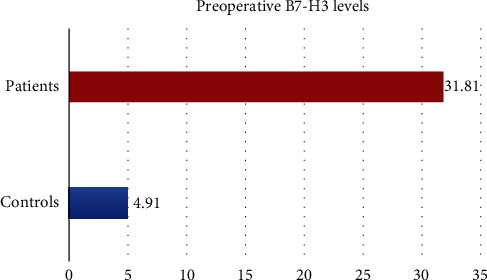
Preoperative B7-H3 levels of the control and patient groups.

The ELISA assay results demonstrated that preoperative serum B7-H3 levels were statistically significantly higher in patients with colon cancer than the healthy control group (*p* < 0.001) (Figure 3).

Preoperative serum B7-H3 concentration of the stage IV patients was 39.81 ± 5.68 ng/mL, with a range of 30-48 ng/mL, and was significantly higher than those of the patients with stage I and stage II disease (Table 2). Likewise, preoperative serum B7-H3 level was significantly higher in patients with a tumor size that was >3 cm, M1, N2, and pT4 stages than patients with a tumor size that was <3 cm, M0, N0, and pT1 (*p* < 0.001). Most importantly, our results demonstrated that B7-H3 concentration may be closely related to aggressive progression of colon cancer.

## 4. Discussion

A large number of studies to date have shown that mortality rate of colon cancer has been declining, which is attributed to early detection through screening and improved treatment [14, 15]. Colon cancer screening is important for the prevention of the occurrence of many cases and detection of colon cancer in early and curable stage; furthermore, regular surveillance after surgery is important for the detection of recurrences without delay, hence improving patients' quality of life and prolonging their life expectancy. Assessment of serum markers may accomplish these goals. Biomarkers play a critical role in early detection and predicting prognosis of a wide range of cancers.

Numerous biomarkers have been investigated for early diagnosis of colon cancer. Many colon cancer markers have the advantage of being noninvasive or minimally invasive, safe, inexpensive, convenient, and easy to obtain among others. Currently, several serum markers such as CEA, CA19-9 (carbohydrate antigen 19-9), CA242 (carbohydrate antigen 242), and TPS (tissue polypeptide specific antigen) are used in colon cancer diagnosis and treatment; however, none of them possesses high sensitivity nor high specificity which would enable their use as screening markers for colon cancer in clinical practice. Actually, these serum markers are rather used as markers of recurrence and prognosis in clinical practice [15–18].

In this study, we investigated the diagnostic value of B7-H3 levels for colon cancer and demonstrated that they had a diagnostic value. Many studies have shown that B7-H3 is involved in cancer progression [8, 19–24]. Studies have shown that adhesion, migration, invasion, and metastasis of cancer cells are influenced by serum B7-H3 levels [25, 26]. In this study, we showed that serum B7-H3 levels have a key role in colon cancer. The molecular mechanisms of the role of B7-H3 remain unclear, and how B7-H3 promotes tumor independently of the immune system is not fully understood. Recently, expression of B7-H3 was shown to inhibit transcription factor NRF2, leading to increased production of reactive oxygen species (ROS) and thus inducing tumor growth [27].

B7-H3 is produced by monocytes, activated T cells, and B7-H3-positive tumor cells [28]. In addition, B7-H3 stimulates VEGF and IL-8 secretion via the TLR4/NF-B pathway, supporting the view that B7-H3 promotes cancer invasion and metastasis. Liu et al. showed that B7-H3 can regulate hepatocellular carcinoma's epithelial to mesenchymal transition by activating Jak2/Stat3 signaling [29]. A study showed a relationship between TGF-*β*1 and B7-H3, indicating that TGF-*β*1 may upregulate B7-H3, leading to classical B7-H3-mediated immune invasion [30]. Perhaps, B7-H3 has an unexpressed role related to the BMP signaling cascade that is involved as a mechanism for B7-H3-induced metastasis. Interestingly, increased B7-H3 expression is correlated to a more aggressive tumor invasion. Our results may also indicate enhanced loading of B7-H3-associated pathogenic molecules such as MMP and chemokines.

In conclusion; we showed that B7-H3 is highly expressed in colon cancer and can be used as a candidate diagnostic biomarker and a potential target in colon cancer in future.

### 4.1. Study Limitations

The major limitation of this study is being conducted in a single center. In addition, the number of participants is relatively small. Finally, further parameters such as follow-up outcomes could be included. However, this study is first to investigate the diagnostic value of preoperative B7-H3 levels as a strength. We believe that our results will be guiding further studies to be performed on the evaluation of B7-H3 in colon cancer.

## Figures and Tables

**Table 1:** Demographic characteristics of the participants.

Parameter	Control group (*n* = 50)		Patient group (*n* = 90)		*p* value
	Mean ± SD	Min–max	Mean ± SD	Min–max	
Age (years)	63.54 ± 2.94	58-69	62.54 ± 3.14	57-69	>0.05
Gender	*n*	%	*n*	%	
(i) Female	25	50	43	47.8	>0.05
(ii) Male	25	50	47	52.2	

SD: standard deviation; min: minimum; max: maximum.

**Table 2:** The clinical stages of the patients.

Parameter	*n*	B7-H3 (ng/mL)		*p* value
		Mean ± SD	Min–max	
<3 cm	12	14.33 ± 0.651	13-15	<0.001
>3 cm	78	34.5 ± 9.25	20-48	
M0	10	14.20 ± 0.632	13-15	
M1	80	34.01 ± 9.63	15-48	
pT1	10	14.20 ± 0.632	13-15	
pT2	13	20.15 ± 2.51	15-23	
pT3	19	26.37 ± 3.77	20-31	
pT4	48	40.79 ± 5.02	30-48	<0.001
N0	10	14.20 ± 0.632	13-15	
N1	13	20.15 ± 2.51	15-23	
N2	67	36.70 ± 8.04	20-48	<0.001
TI	11	14.27 ± 0.647	13-15	
TII	13	20.77 ± 2.088	15-23	
TIII	13	25.08 ± 3.42	20-30	
TIV	53	39.81 ± 5.68	30-48	<0.001

SD: standard deviation; min: minimum; max: maximum.

## Data Availability

Data used in this study can be provided on reasonable request.
